# Effect of Repetitive Peripheral Magnetic Stimulation in Patients with Neck Myofascial Pain: A Randomized Sham-Controlled Crossover Trial

**DOI:** 10.3390/jcm14155410

**Published:** 2025-08-01

**Authors:** Thapanun Mahisanun, Jittima Saengsuwan

**Affiliations:** 1 Department of Rehabilitation Medicine, Thabo Crown Prince Hospital, Nongkhai 43110, Thailand; thapanun84@gmail.com; 2 Department of Rehabilitation Medicine, Faculty of Medicine, Khon Kaen University, Khon Kaen 40002, Thailand

**Keywords:** peripheral magnetic stimulation, myofascial pain syndrome

## Abstract

**Background/Objectives**: Neck pain caused by myofascial pain syndrome (MPS) is a highly prevalent musculoskeletal condition. Repetitive peripheral magnetic stimulation (rPMS) is a promising treatment option; however, its therapeutic effect and optimal treatment frequency remain unclear. This study aimed to investigate the therapeutic effect and duration of effect of rPMS in patients with MPS of the neck. **Methods**: In this randomized, sham-controlled, crossover trial, 27 patients with neck MPS and baseline visual analog scale (VAS) scores ≥ 40 were enrolled. The mean age was 43.8 ± 9.1 years, and 63% were female. Participants were randomly assigned to receive either an initial rPMS treatment (a 10 min session delivering 3900 pulses at 5–10 Hz) or sham stimulation. After 7 days, groups crossed over. Pain intensity (VAS), disability (Neck Disability Index; NDI), and analgesic use were recorded daily for seven consecutive days. A linear mixed-effects model was used for analysis. **Results**: At baseline, the VAS and NDI scores were 61.8 ± 10.5 and 26.0 ± 6.3, respectively. rPMS produced a significantly greater reduction in both VAS and NDI scores, with the greatest differences observed on Day 4: the differences were −24.1 points in VAS and −8.5 points in NDI compared to the sham group. There was no significant difference in analgesic use between the two groups. **Conclusions**: A single rPMS session provides short-term improvement in pain and disability in neck MPS. Based on the observed therapeutic window, more frequent sessions (e.g., twice weekly) may provide sustained benefit and should be explored in future studies.

## 1. Introduction

According to the Global Burden of Disease Study, neck pain is a highly prevalent condition, and the global number of cases is projected to increase from 2020 to 2050 due to population growth and aging [1]. Neck pain is also a significant cause of disability, ranking 11th out of 369 conditions in terms of years lived with disability (YLDs) in a recent analysis [1].

Myofascial pain syndrome (MPS) is one of the most common causes of neck pain. Previous studies have reported that between 88.9% and 100% of patients with nonspecific chronic neck pain have at least one myofascial trigger point [2,3], with the upper trapezius muscle being the most frequently affected site [3]. Several risk factors have been proposed for the development of MPS, including muscle overuse, postural imbalance, vitamin deficiencies, chronic stress, and a sedentary lifestyle [4]. The primary objectives of myofascial pain syndrome (MPS) treatment are to relieve pain, reduce muscle tension, and deactivate trigger points [5]. Effective management often requires a multimodal approach that incorporates both pharmacological and non-pharmacological interventions [4]. Non-pharmacological treatments are generally classified as either non-invasive or invasive. Non-invasive methods include cold spray therapy, ultrasound therapy, muscle stretching, massage, and transcutaneous electrical nerve stimulation (TENS), while invasive treatments involve procedures such as dry needling, acupuncture, or trigger point injection [6,7].

Peripheral magnetic stimulation (PMS) is a non-invasive and well-tolerated therapeutic modality. It generates high-intensity magnetic pulses through coils to stimulate muscles and sensorimotor nerve fibers [4]. Previous studies have demonstrated the effectiveness of PMS in relieving various types of pain, including acute low back pain [8], neuropathic pain from traumatic brachial plexopathy [9], and myofascial pain syndrome (MPS) [10,11].

Although the exact mechanisms underlying PMS-induced pain relief remain to be fully elucidated, several theories have been proposed. Repetitive peripheral magnetic stimulation (rPMS) may recruit peripheral and proprioceptive afferents by directly activating sensorimotor nerve fibers and indirectly stimulating mechanoreceptors within muscle tissue [12]. It may also modulate cortical excitability by balancing excitation and inhibition in the targeted muscles, enhancing both the timing and intensity of muscle activation. This neuromodulatory effect may help normalize abnormal postural adjustment patterns [13]. Moreover, rPMS may modulate central pain pathways by affecting nociceptive afferent transmission in the brainstem, thus influencing central mechanisms of pain perception [14].

Randomized controlled trials have reported significant improvements in both pain and cervical range of motion (ROM) in patients with upper trapezius MPS following rPMS treatment [10,11]. Banyat N. compared the effectiveness of PMS and dry needling in patients with cervical MPS. Participants received PMS once a week for one month, using a frequency of 5 Hz during the scanning phase and 10–20 Hz during the therapeutic phase, with intensity adjusted individually. The study found that PMS reduced pain and improved functional activity levels comparably to dry needling [15]. Similarly, Pujol et al. reported that PMS was more effective than sham treatment in reducing localized musculoskeletal pain, with pain relief lasting for several days [16]. However, in that study, some patients received only one rPMS session, while others received two sessions within a one-week period, making it difficult to determine the exact duration of the treatment effect. To date, no study has specifically investigated the duration of the therapeutic effects of PMS in patients with neck MPS. Therefore, the aim of this study was to evaluate both the therapeutic efficacy and the duration of effect of PMS in individuals with myofascial pain syndrome of the neck.

## 2. Materials and Methods

This study was a randomized, sham-controlled, crossover trial. We enrolled 27 patients with myofascial pain syndrome (MPS) of the neck who were treated at the outpatient department of Thabo Crown Prince Hospital between November 2023 and March 2024. The diagnosis of MPS was confirmed by a physiatrist based on the presence of five major criteria: localized pain, presence of a taut band, a tender spot within the taut band, a characteristic referred pain pattern, and some degree of restricted range of motion in the affected muscle. Additionally, at least one minor criterion had to be present: reproduction of clinical symptoms upon compression of the active trigger point, elicitation of a local twitch response upon palpation, and the alleviation of pain following muscle stretching [17]. The inclusion criteria were as follows: (1) age between 18 and 60 years; (2) neck pain lasting more than three months; (3) a pain score of ≥40 on the visual analog scale (VAS); and (4) no prior history of PMS treatment. Exclusion criteria included the following: a concomitant diagnosis of non-mechanical neck pain (e.g., ankylosing spondylosis or previous cervical spine injury), severe psychiatric disorders, use of a pacemaker or other implanted electronic/metal devices, prior cervical spine surgery, and the presence of cancer or tumors in the neck region.

Ethical approval for this study was obtained from the Institutional Review Board of the Nongkhai Provincial Public Health Office (IRB number: 87/2567). The trial was also prospectively registered with the Thai Clinical Trials Registry (TCTR), an online registry of clinical research in Thailand (thaiclinicaltrials.org under the identifier TCTR20241110004). Written informed consent was obtained from all participants prior to their enrollment in this study.

### 2.1. Procedures

**Figure 1:**
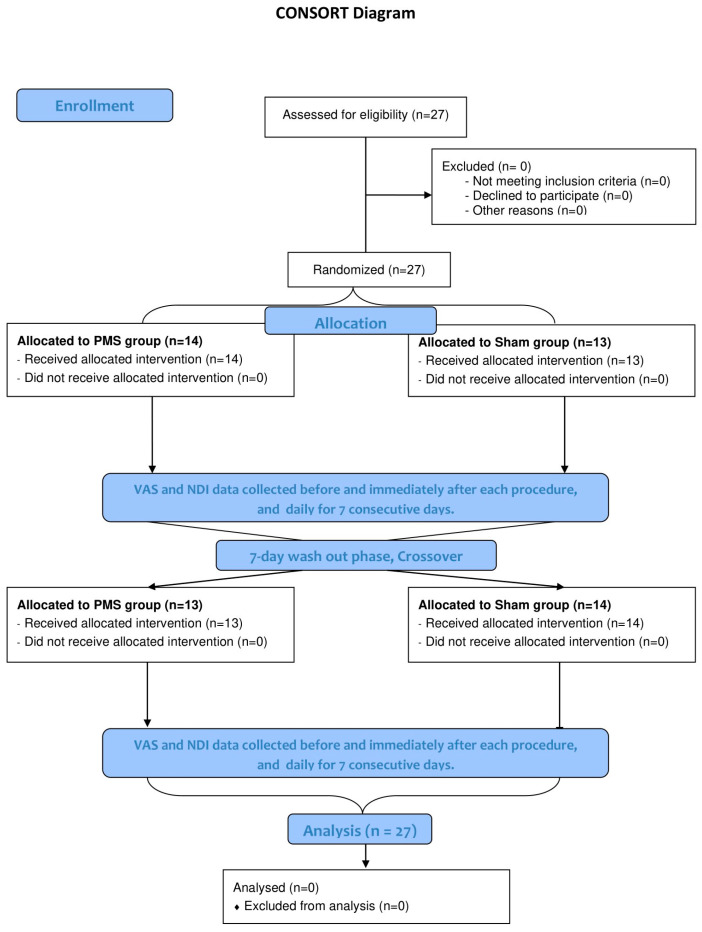
Flow diagram of the study.

Participants were randomly assigned to the study groups using block randomization (block size of four), with allocation concealment ensured through sealed, opaque envelopes. The randomization process was conducted by a pharmacist who was not involved in any other aspect of this study. Fourteen participants were initially allocated to the PMS group, and thirteen were allocated to the sham group. After a seven-day period, the groups were crossed over to receive the alternate intervention (Figure 1).

In the PMS group, participants received rPMS treatment using a circular coil (Salus Talent Pro model, REMED Co., Ltd., Seoul, Republic of Korea) applied directly to the identified trigger point area. The stimulation intensity began at 20% of the maximal stimulator output and was gradually increased in 5% increments until visible muscle contractions were observed. The treatment lasted 10 min and was delivered in three phases:

1. Scan phase: 5 Hz continuous stimulation for 3 min (total of 900 pulses).
2. Therapeutic phase: 10 Hz stimulation with a train duration of 4 s and an intertrain interval of 1 s (total of 2400 pulses) over 5 min. The intensity was adjusted to produce visible muscle contractions while ensuring patient comfort.
3. Cool down phase: 5 Hz continuous stimulation for 2 min (total of 600 pulses).

In the sham group, treatment was administered in the same manner, but the coil was positioned perpendicular to the skin surface. However, patients still heard the rhythmic sound of the device, similar to the active treatment group, to maintain blinding.

Both groups also received standard conservative treatment, including patient education on ergonomic practices and neck stretching exercises. Participants were instructed to perform 10 repetitions per set, twice daily.

### 2.2. Outcome Measurement

The primary outcome measures included the visual analog scale (VAS) and the Neck Disability Index (NDI), Thai Version. The VAS is a 100-point scale used to assess pain intensity, where 0 represents no pain and 100 represents the worst pain imaginable [18]. Physical disability related to neck pain was assessed using NDI, which consists of 10 items. Each item is scored from 0 to 5, with a total possible score ranging from 0 to 50. A score of 0 indicates no limitations in performing activities, while a score of 5 indicates complete limitation. The Thai version of the NDI has excellent reliability, with a test–retest reliability of 0.986 and a Cronbach’s alpha for internal consistency of 0.925 [19].

VAS and NDI data were collected through interviews before and immediately after the procedure. Additionally, participants were asked to record their VAS and NDI scores once daily, at the same time each day, for seven consecutive days following the intervention. Daily medication intake was also recorded once a day. The interviews and assessments were carried out by the same physical therapist, who was blinded to group allocation. Participants were permitted to use only diclofenac (50 mg) for pain relief during the study period, and the daily dosage intake was recorded throughout the seven-day follow-up.

### 2.3. Sample Size Calculation

The sample size was calculated using a formula for crossover studies with continuous outcomes [20]. The minimally clinically important difference (MCID) and standard deviation (SD) of the VAS score were 16.55 and 17.5, respectively [21]. However, to account for potentially greater variability in patient responses within our study population, we increased the SD by approximately 50% to 27. This adjustment was made to minimize the risk of underpowering the study. A significance level (α) of 0.05 and a power (1−β) of 0.8 were used. Based on these parameters, the required sample size was 21 participants per group. To allow for an anticipated 20% attrition rate, the final sample size was increased to 27 participants per group.

### 2.4. Statistical Analysis

Quantitative data were presented as mean and standard deviation for normally distributed data, and as median, 25th, and 75th percentile for non-normally distributed data. Categorical data were summarized using frequency and percentage.

This study employed an AB/BA crossover design with two periods and two treatments. To assess potential carryover or sequence effects, a two-way ANOVA for a 2 × 2 crossover design was conducted. All analyses were performed under the intention-to-treat principle. A linear mixed model using restricted maximum likelihood estimation was employed, with treatment group and time as fixed effects and subject as a random effect, to estimate the group effects, time effects, and group × time interactions.

A *p*-value of < 0.05 was considered statistically significant. All statistical analyses were performed using Stata version 18 (Stata Corp, College Station, TX, USA).

## 3. Results

A total of 27 patients with myofascial pain syndrome (MPS) of the neck were enrolled in this study. The majority of participants were female (63.0%), with a mean age of 43.8 ± 9.1 years. The mean body mass index (BMI) was 24.5 ± 2.6 kg/m^2^. Among these patients, 14.8% had underlying comorbidities, including hypertension (7.4%), type 2 diabetes mellitus (3.7%), and dyslipidemia (3.7%). The median duration of neck pain was 12 months. More than half (55.6%) had previously received medication for pain, while 40.7% reported other co-treatments such as massage (25.9%) and physical therapy (14.8%). All identified trigger points were located in the upper trapezius muscle (Table 1).

**Figure 2:**
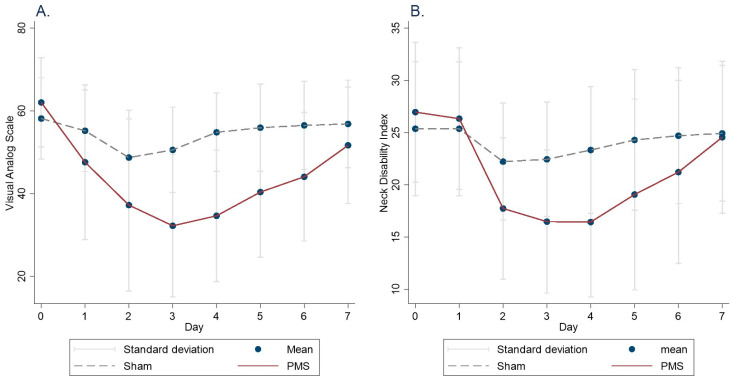
Changes in (**A**) VAS and (**B**) NDI from Day 0 to Day 7.

The mean baseline visual analog scale (VAS) was 61.8 ± 10.5, and the mean Neck Disability Index (NDI) score was 26.0 ± 6.3. The treatment effects of PMS and sham interventions are summarized in Table 2 and illustrated in Figure 1. No significant carryover or sequence effects were detected. Analysis using a linear mixed-effects model revealed a significant time effect, with reductions in both VAS and NDI scores on Days 2 and 3 in both the PMS and sham groups. There was no significant main effect of treatment group alone. However, a significant interaction effect between treatment and time was identified. Specifically, the PMS group showed a significantly greater reduction in VAS score from Day 1 to Day 7 compared to the sham group, with the greatest difference observed on Day 4 (−24.1 points compared to sham). Similarly, a significantly greater reduction in NDI score was observed from Day 1 to Day 6, with the greatest improvement in disability on Day 4 (−8.5 points compared to sham) (Table 2, Figure 2). There was no significant difference in diclofenac use between the two groups (Table 2).

## 4. Discussion

This study aimed to evaluate the therapeutic effects and duration of action of repetitive peripheral magnetic stimulation (rPMS) in patients with myofascial pain syndrome (MPS) of the neck. Specifically, we compared the efficacy of PMS combined with conservative treatment to sham stimulation combined with the same conservative management. The PMS intervention targeted the identified pain areas with a total stimulation time of 10 min per session, delivering magnetic pulses at frequencies of 5–10 Hz (totaling 3900 pulses per session).

Our findings demonstrated that rPMS significantly reduced pain and improved functional disability in patients with neck MPS when compared to the sham group. A noticeable reduction in pain was observed as early as the first day following treatment, as reflected by a decrease in the visual analog scale (VAS) score. These findings are consistent with previous studies reporting immediate analgesic effects of PMS in various musculoskeletal pain conditions [10,11,22,23].

Regarding stimulation parameters, there are currently no standardized protocols for rPMS in the management of musculoskeletal pain. Previous studies have used stimulation frequencies ranging from 5 to 20 Hz and pulse counts between 600 and 4500, with higher frequencies generally considered more effective for sensorimotor nerve activation [15,16,24]. Based on this evidence, we selected a frequency of 5–10 Hz and a total of 3900 pulses per session. The treatment was well tolerated, and no adverse events were reported.

According to the study by Young et al., the minimum clinically important difference (MCID) for the NDI and numeric pain rating scale (NRS, 0–10) in patients with mechanical neck pain is 5.5 and 1.5 points, respectively [25]. Translating this to our 0–100 VAS scale, a difference exceeding 15 points would be considered clinically meaningful. In our findings, the mean reduction in VAS scores in the PMS group exceeded 15 points from Day 2 through Day 6, but not on Day 7, suggesting a waning effect. Additionally, while the same group did not achieve an MCID in NDI on any day, the PMS group exceeded the MCID threshold from Day 2 to Day 6. Interestingly, both pain and functional improvements peaked around Days 3 and 4, with the PMS group experiencing a nearly 40% reduction in pain by Day 4. These findings align with studies in knee osteoarthritis, where significant improvements were observed immediately following rPMS but diminished by the seventh day post-treatment.

A key objective of this study was to investigate the duration of therapeutic effects, as this may guide clinical decisions regarding the optimal frequency of treatment sessions. Existing literature on rPMS for musculoskeletal pain varies in treatment frequency, ranging from once weekly to five times per week [10,11,14,22]. In our study, we evaluated changes in VAS and NDI scores for seven consecutive days following a single treatment session. Based on our data, a once-weekly session may not be sufficient to sustain the clinical benefit. Therefore, more frequent sessions—such as biweekly—may be more appropriate for maintaining therapeutic gains in this patient population.

This study has several limitations. First, the sample size was recruited from a single center, which may limit the generalizability of the findings. Second, although we employed a crossover design to control for inter-individual variability, the short washout period may not have fully eliminated carryover effects, despite statistical analysis showing no significant carryover. Third, the duration of follow-up was limited to seven days, which does not allow conclusions about the long-term efficacy or optimal treatment frequency of PMS. Fourth, this study focused only on a working-age population (18–60 years), excluding older adults and adolescents, who may respond differently to PMS. Fifth, the use of only one PMS protocol (frequency, intensity, and duration) prevents exploration of dose–response relationships or optimization of stimulation parameters. Sixth, although participants and assessors were blinded, maintaining complete blinding in a crossover design involving PMS is challenging due to the physical sensations associated with active stimulation. This potential unblinding may have introduced bias, especially since outcomes were primarily self-reported using measures such as the VAS and NDI. Additionally, the lack of monitoring for adherence to home-based exercises could have contributed to variability between groups and influenced the results. Future studies should include a larger and more diverse population, incorporate blinding assessments, explore alternative study designs, and test multiple PMS protocols and longer follow-up periods to determine the most effective treatment regimen and long-term benefits. Incorporating objective measures, such as cervical range of motion or electromyography, along with tracking participants’ adherence to home exercises, may further enhance the validity and reliability of the findings.

In summary, PMS is an effective, safe, and well-tolerated intervention for patients with neck myofascial pain syndrome. It provides significant pain relief and functional improvement, with peak effects observed on the third to fourth day following treatment and maintained up to the sixth day. To optimize patient outcomes, the frequency of treatment sessions should be carefully considered. Determining the optimal schedule can help ensure sustained therapeutic benefits while minimizing unnecessary treatment sessions, reducing patient travel burdens, and lowering healthcare costs.

## 5. Conclusions

A single session of peripheral magnetic stimulation delivering 3900 pulses over 10 min provides significant short-term pain relief in patients with neck myofascial pain syndrome compared to sham treatment. Maximum pain reduction was observed on the third and fourth days post-treatment, with effects sustained up to the sixth day.

## Figures and Tables

**Table 1:** Demographic data of participants (n = 27).

Variables	N (%)
Male/Female	10 (37.0)/17 (63.0)
Age (years), mean (SD)	43.8 (9.1)
Marital status	
Married	16 (59.3)
Single	5 (18.5)
Divorce/Widow/Separated	6 (32.2)
Educational level	
Lower or equal to secondary school	18 (66.7)
Higher than secondary school	9 (33.3)
Body mass index (kg/m^2^), mean (SD)	24.5 (2.6)
Smoking	3 (11.1)
Alcoholic drinking	8 (29.6)
Underlying disease	
Hypertension	2 (7.4)
Diabetes Mellitus	1 (3.7)
Dyslipidemia	1 (3.7)
Duration of neck pain (months), median (p25, p75)	12 (4, 12)
Baseline VAS, mean (SD)	61.8 (10.5)
Baseline NDI, mean (SD)	26.0 (6.3)
Pain medication	15 (55.6)
NSAIDs	6 (22.2)
Muscle relaxant	4 (14.8)
Acetaminophen	10 (37.0)
Concomitant treatment	
Physical therapy	4 (14.8)
Massage	7 (25.9)

Data are presented in n (%) unless otherwise specified.

**Table 2:** Changes in VAS and NDI in each group and mean differences between groups over time (N = 27).

Variables	Sham Group Mean (95% CI)	PMS Group Mean (95% CI)	Adjusted Differences (95% CI) *	*p*-Value *
**VAS**				
Pretreatment	58.3 (54.6–61.9)	61.9 (57.7–66.2)		
Day 1	55.3 (51.5–59.0)	47.5 (40.9–54.1)	−11.5 (−16.7 to −6.3)	<0.001
Day 2	48.8 (44.4–53.1)	37.1 (29.9–44.3)	−15.4 (−20.7 to −10.1)	<0.001
Day 3	50.7 (46.7–54.6)	32.1 (25.8–38.4)	−22.2 (−27.7 to −16.8)	<0.001
Day 4	54.9 (51.4–58.4)	34.5 (28.7–40.4)	−24.1 (−29.7 to −18.4)	<0.001
Day 5	56.0 (52.1–59.9)	40.3 (34.5–46.0)	−19.4 (−25.3 to −13.6)	<0.001
Day 6	56.6 (52.2–60.7)	44.0 (38.5–49.4)	−16.3 (−22.5 to −10.1)	<0.001
Day 7	57.0 (53.0–60.9)	51.6 (46.6–56.6)	−9.1 (−15.5 to −2.6)	<0.001
**NDI**				
Pretreatment	25.3 (23.0–27.8)	26.9 (24.3–29.5)		
Day 1	25.4 (23.0–27.8)	26.3 (23.7–28.9)	−0.6 (−2.6 to 1.3)	0.53
Day 2	22.2 (20.1–24.4)	17.7 (15.2–20.2)	−6.1 (−8.1 to −4.1)	<0.001
Day 3	22.5 (20.4–24.5)	16.5 (13.9–19.1)	−7.6 (−9.6 to −5.5)	<0.001
Day 4	23.3 (21.1–25.6)	16.4 (13.7–19.1)	−8.5 (−10.6 to −6.4)	<0.001
Day 5	24.3 (21.8–26.8)	19.1 (15.6–22.5)	−6.8 (−9.0 to −4.6)	<0.001
Day 6	24.7 (22.3–27.2)	21.2 (17.9–24.5)	−5.1 (−7.3 to −2.8)	<0.001
Day 7	24.9 (22.5–27.4)	24.5 (21.8–27.3)	−2.0 (−4.3 to 0.4)	0.11
**Diclofenac used** **(Tabs** **)**				
Day 1	0.00 (−0.03–0.04)	−0.0 (−0.17–0.01)	−0.01 (−0.05 to 0.03)	0.70
Day 2	0.04 (−0.03–0.12)	0.07 (−0.07–0.21)	0.02 (−0.13 to 0.18)	0.76
Day 3	0.00 (−0.03–0.04)	0.03 (−0.04–0.10)	0.03 (−0.05 to 0.10)	0.48
Day 4	0.20 (−0.11–0.50)	0.07 (−0.08–0.21)	−0.13 (−0.48 to 0.22)	0.46
Day 5	0.24 (−0.09–0.56)	0.07 (−0.03–0.16)	−0.17 (−0.52 to 0.18)	0.34
Day 6	0.08 (−0.03–0.19)	0.21 (−0.07–0.49)	0.12 (−0.17 to 0.42)	0.39
Day 7	0.20 (−0.02–0.41)	0.24 (−0.05–0.54)	0.05 (−0.31 to 0.41)	0.78

* Analysis was conducted using a linear mixed model for repeated measures with restricted maximum likelihood estimation. The table shows the group x time interaction and corresponding *p*-values.

## Data Availability

The original contributions presented in this study are included in this article. Further inquiries can be directed to the corresponding author.
